# Cascaded Amplitude Modulations in Sound Texture Perception

**DOI:** 10.3389/fnins.2017.00485

**Published:** 2017-09-11

**Authors:** Richard McWalter, Torsten Dau

**Affiliations:** Hearing Systems Group, Technical University of Denmark Kongens Lyngby, Denmark

**Keywords:** sound texture, amplitude modulation, auditory model, natural sound, auditory perception

## Abstract

Sound textures, such as crackling fire or chirping crickets, represent a broad class of sounds defined by their homogeneous temporal structure. It has been suggested that the perception of texture is mediated by time-averaged summary statistics measured from early auditory representations. In this study, we investigated the perception of sound textures that contain rhythmic structure, specifically second-order amplitude modulations that arise from the interaction of different modulation rates, previously described as “beating” in the envelope-frequency domain. We developed an auditory texture model that utilizes a cascade of modulation filterbanks that capture the structure of simple rhythmic patterns. The model was examined in a series of psychophysical listening experiments using synthetic sound textures—stimuli generated using time-averaged statistics measured from real-world textures. In a texture identification task, our results indicated that second-order amplitude modulation sensitivity enhanced recognition. Next, we examined the contribution of the second-order modulation analysis in a preference task, where the proposed auditory texture model was preferred over a range of model deviants that lacked second-order modulation rate sensitivity. Lastly, the discriminability of textures that included second-order amplitude modulations appeared to be perceived using a time-averaging process. Overall, our results demonstrate that the inclusion of second-order modulation analysis generates improvements in the perceived quality of synthetic textures compared to the first-order modulation analysis considered in previous approaches.

## Introduction

Sound textures are characterized by their temporal homogeneity and may be represented with a relatively compact set of time-averaged summary statistics measured from early auditory representations (Saint-Arnaud and Popat, 1995; McDermott et al., 2013). Although, textures can be expressed in a relatively compact form, they are ubiquitous in the natural world and span a broad perceptual range (e.g., rain, fire, ocean waves, insect swarms etc.). The perceptual range has been defined by a set of *texture* statistics outlined by McDermott and Simoncelli (2011). However, it remains unclear what sound features might also be represented in the auditory system via a time-averaging mechanism. In the present study, we investigated and expanded the perceptual space of texture, particularly in the domain of amplitude modulations.

The texture synthesis system of McDermott and Simoncelli (2011) described spectral and temporal tuning properties of the early auditory system that are crucial for texture perception. Synthetic textures were generated by measuring time-averaged texture statistics at the output of several processing stages of a biologically plausible auditory model, which were subsequently used to shape a Gaussian noise seed to have matching statistics. The auditory texture model included frequency-selective auditory filters and amplitude-modulation selective filters derived from both psychophysical and physiological data (Dau et al., 1997). The authors demonstrated that when the auditory model deviated in its biological plausibility, such as applying linearly spaced auditory filters, the perceptual quality of the texture exemplars was reduced. In addition, McDermott and Simoncelli (2011) identified which texture statistics were necessary for correct identification, revealing subsets of statistics that were requisite for different sound textures. Collectively, the results suggested that textures synthesized with the complete set of texture statistics and a biologically plausible auditory model were preferred over all other identified synthesis system configurations.

The sound synthesis system proposed by McDermott and Simoncelli (2011) generated compelling exemplars for a broad range of sounds, but there were also sounds for which the auditory texture model failed to capture some of the perceptually significant features. The failures were identified by means of a realism rating performed by human listeners, who compared synthetic textures to corresponding original real-world texture recordings. The shortcomings were attributed to either the processing structure or the statistics measured from the auditory texture model. One such texture group were sounds that contained rhythmic structure (McDermott and Simoncelli, 2011).

In the present study, the auditory texture model of McDermott and Simoncelli (2011) was extended to include sensitivity to second-order amplitude modulations. Second-order amplitude modulations arise from beating in the envelope-frequency domain. Intuitively, this can be described as the interaction between two modulators acting on a carrier. At slow modulation rates, second-order amplitude modulations have the perceptual quality of simple rhythms (Lorenzi et al., 2001a). This type of amplitude modulation has been shown to be salient in numerous behavioral experiments (Lorenzi et al., 2001a,b; Ewert et al., 2002; Verhey et al., 2003; Füllgrabe et al., 2005). The perception of second-order amplitude modulation has also been modeled by applying non-linear processing and modulation-selective filtering to a signal's envelope (Ewert et al., 2002). While the role of second-order amplitude modulation in sound perception has been investigated using artificial stimuli, their significance in natural sound perception has yet to be examined.

**Figure 1:**
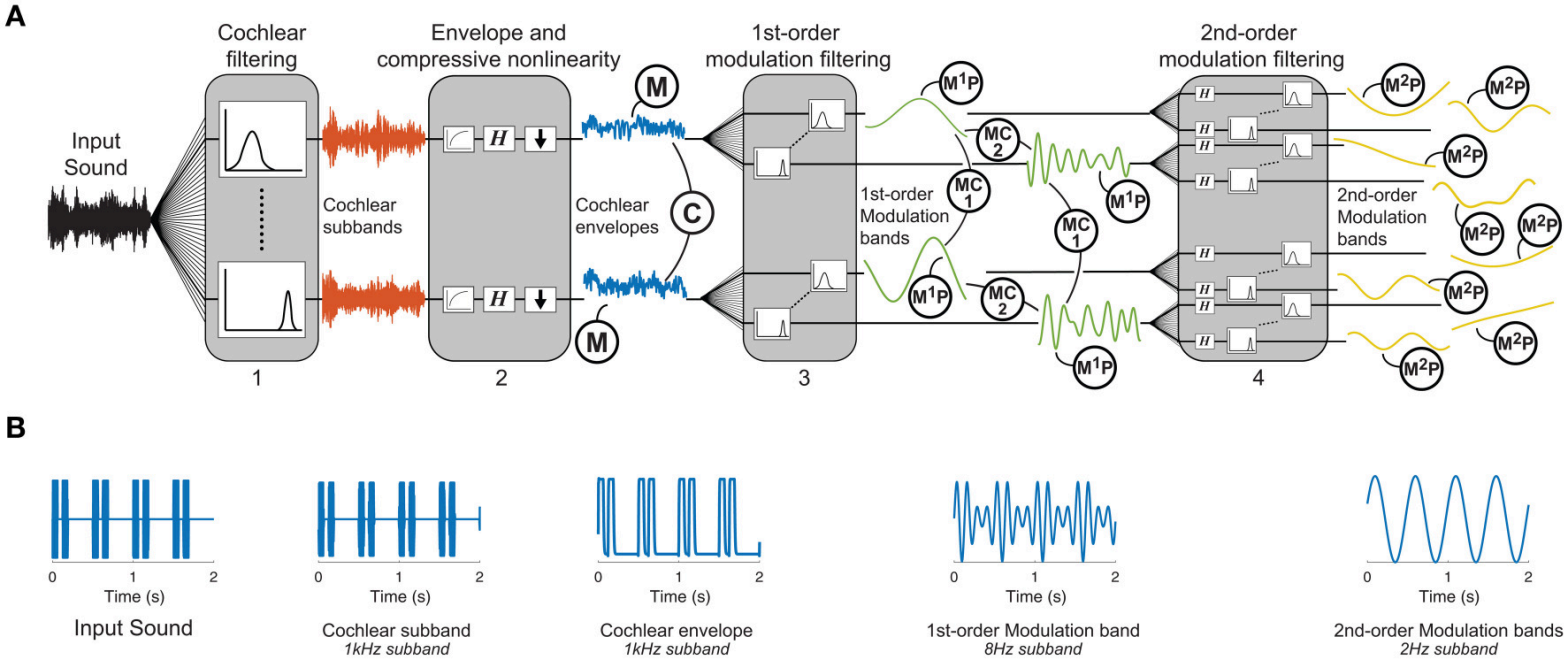
Texture analysis model. **(A)** The functional auditory model captures the tuning properties of the peripheral and subcortical auditory system: (1) An auditory filterbank simulates the resonance frequencies of the cochlea, (2) a non-linearity captures the compression of the cochlea followed by a computation of the Hilbert envelope, functionally modeling the transduction from the mechanical vibrations on the basilar membrane to the receptor potentials in the hair cells, (3) a first-order modulation filterbank captures the selectivity of the auditory system to different envelope fluctuation rates, and (4) a second-order modulation filterbank captures the sensitivity of the auditory system to beating in the envelope frequency domain. Texture statistics include marginal moments of cochlear envelopes (M), 1st-order modulation power (M^1^P), pair-wise correlations between cochlear envelopes (C), pairwise correlations between modulation subbands (MC_1_), phase correlations between octave-spaced modulation bands (MC_2_), and 2nd-order modulation power (M^2^P). **(B)** Example second-order modulation stimulus. The far-left panel shows the input stimulus that consists of two short 62.5 ms pulses repeated every 500 ms. The example outputs are shown at each stage of the model. The output of the 1st-order modulation band is shown for the 8 Hz subband which captures the period of the short pulses. The 2nd-order modulation band is shown for the 2 Hz subband which captures the period of the repetition.

We undertook an analysis-via-synthesis approach to examine the role of second-order amplitude modulations in sound texture perception (Portilla and Simoncelli, 2000; McDermott and Simoncelli, 2011). This entailed generating synthetic sounds from time-averaged statistics measured at different stages of our auditory texture model (Figure 1A). The synthetic sounds were controlled by two main factors: the structure of the auditory texture model and the statistics passed to the texture synthesis system. We first ensured that the sound texture synthesis system was able to capture the temporal structure of a second-order amplitude modulated signal (Figure 1B). Subsequently, we examined the significance of the auditory texture model in a series of behavioral texture identification and preference tasks. Lastly, we attempted to quantify the role of time-averaging in the perception of second-order amplitude modulation stimuli.

## Methods

### Auditory texture model

The auditory texture model is based on a cascaded filterbank structure that separates the signal into frequency subbands (Figure 1A). The first stage of the model uses 34 gammatone filters, equally spaced on the equivalent rectangular bandwidth (ERB)_N_ scale from 50 Hz to ~8 kHz (Glasberg and Moore, 1990):

$$g\left(\;t\right)=\;ct^{3}e^{2\pi i\cdot \;f_{c}\cdot \;t}e^{-\;2\pi \cdot \beta \cdot \;t},$$

where *f*_*c*_ is the gammatone center frequency, β is a bandwidth tuning parameter and *c* is a scale coefficient. Although gammatone filters only capture the basic frequency selectivity of the auditory system, more advanced and dynamic filterbank architectures, such as dynamic compressive gammachirp filters (Irino and Patterson, 2006), did not yield any improvement in texture synthesis as observed in pilot experiments. To allow for the reconstruction of the subbands, a paraconjugate filter, $\tilde{G}\left(z\right)$, was created for each gammatone filter, *G*(*z*) (Bolcskei et al., 1998):

$$\tilde{G}\left(\;z\right)=\left(\frac{1}{G\left(z\right)}\right)\cdot \left(G\left(\;z\right)G{\left(z\right)}^{T}+\;G^{*}\left(\;z\right)G^{*}{\left(\;z\right)}^{T}\right),$$

where *G*(*z*) is the Fourier transform of *g*(*t*), and G^*^(*z*) is the complex conjugate of *G*(*z*). Perfect reconstruction is achieved as long as:

$$\tilde{G}\left(\;z\right)G\left(\;z\right)=\;1.$$

To model fundamental properties of the peripheral auditory system, we applied compression and envelope extraction to the subband signals. The compression was used to model the non-linear behavior of the cochlea (e.g., Ruggero, 1992) and was implemented as a power-law compression with an exponent value of 0.3. As all textures were presented at a sound pressure level (SPL) of 70 dB, it was deemed not necessary to include level-dependent compression. To functionally model the transduction from the cochlear to the auditory nerve, the envelopes of the compressed subbands were extracted using the Hilbert transform and down-sampled to 400 Hz (McDermott and Simoncelli, 2011). The compressed, down-sampled envelopes roughly estimate the transduction from basilar-membrane vibrations to inner hair-cell receptor potentials.

The model then processed each cochlear channel signal by a modulation filterbank, accounting for the first-order modulation sensitivity and selectivity of the auditory system. The filterbank applied to each cochlear channel comprised of 19 filters, half-octave spaced from 0.5 to 200 Hz. This type of functional modeling is consistent with previous perceptual models of modulation sensitivity (Dau et al., 1997) and shares similarities with neurophysiological findings (Miller et al., 2002; Joris et al., 2004; Malone et al., 2015). The broadly tuned modulation filters have a constant *Q* = 2 and a shape defined by a Kaiser–Bessel window. Reconstruction of the modulation filterbank was achieved with the same method as the frequency selective gammatone filterbank.

The output of each modulation filter was subsequently processed by a second modulation filterbank, accounting for the sensitivity of the auditory system to second-order amplitude modulations. Each second-order modulation filterbank contained 17, half-octave spaced bands in the range from 0.25 to 64 Hz. The model was inspired by behavioral experiments and simulations revealing an auditory sensitivity to second-order modulations that is similar in nature to the sensitivity to first-order amplitude modulations (Lorenzi et al., 2001a,b; Ewert et al., 2002; Füllgrabe et al., 2005). The model processing layer proposed here has some shared attributes to the model presented in Ewert et al. (2002), but has the added benefit of being easily invertible. The second-order modulation filters have a constant *Q* = 2 and a Kaiser–Bessel window.

### Texture statistics

The goal of statistics selection is to find a description of sound textures that is consistent with human sensory perception (Portilla and Simoncelli, 2000). The selected statistics should be based on relatively simple operations that could plausibly occur in the neural domain. The values of the measured statistics should also vary across textures, facilitating the recognition of sound textures by the difference in the statistical representation. Lastly, there should be a perceptual salience to the textures, such that the use of their statistics contributes to the realism of the corresponding synthetic texture.

**Figure 2:**
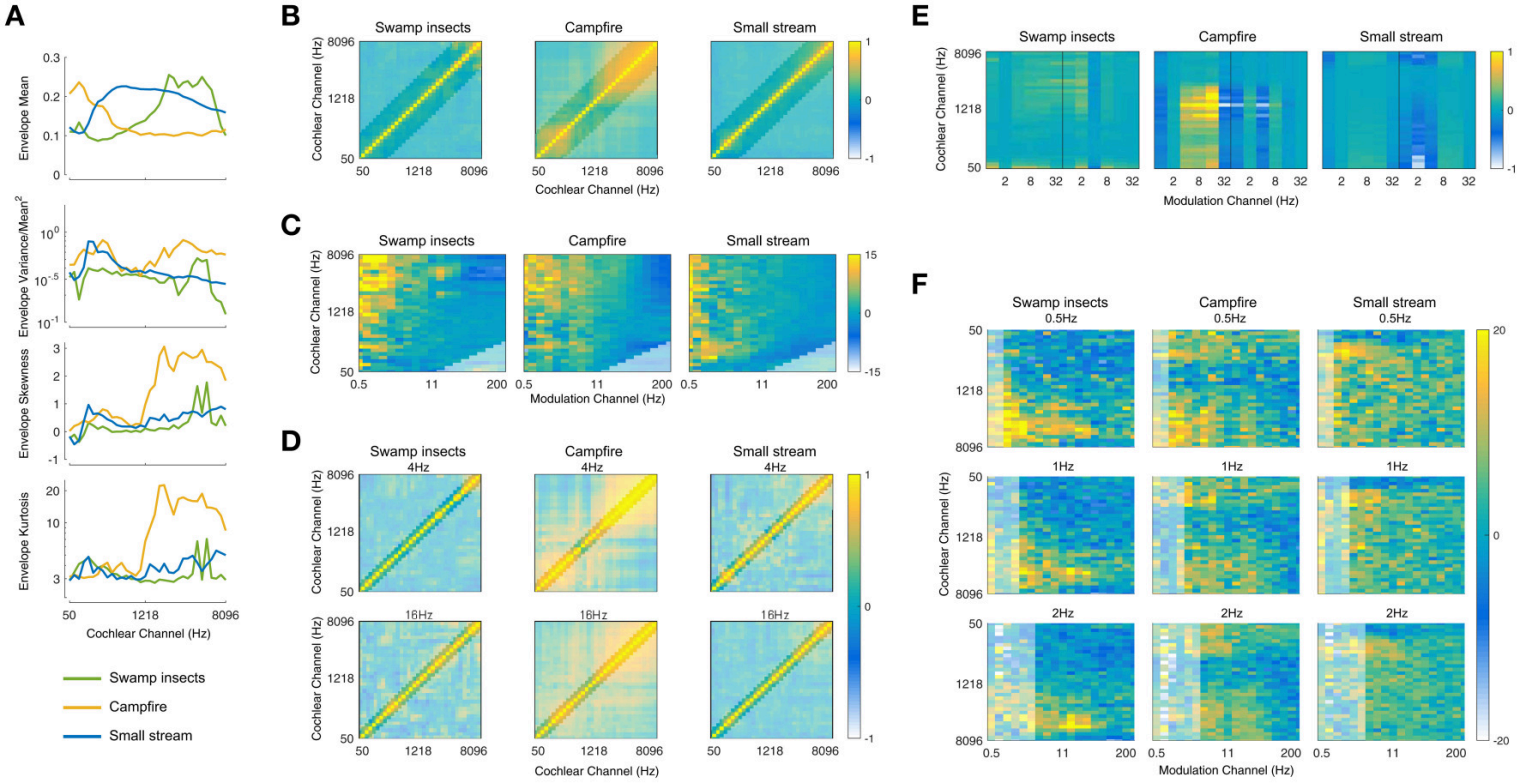
Texture Statistics. **(A)** Cochlear envelope marginal moments (mean, coefficient of variance, skewness, kurtosis) measured from three real-world texture recordings (Swamp insects, campfire, small stream). **(B)** Cochlear envelope pair-wise correlations measured between different cochlear channels. The label of the texture analyzed is located above the subfigure (and for all subsequent subfigures). Lightened regions here and elsewhere denote texture statistics that are not imposed during the synthesis process. **(C)** Modulation band power (variance). The figure is normalized by the modulation power of Gaussian noise and shown on a log (dB) scale. **(D)** Modulation correlation measured for a particular rate across cochlear channels. The modulation rate is indicated above the subfigure. **(E)** Modulation phase correlation measured between octave-spaced modulation bands. **(F)** Second-order modulation band power (variance). The second-order modulation frequency is indicated above the individual subfigures for a selection of rates (0.5, 1, and 2 Hz). The statistics are plotted relative to Gaussian noise on a log (dB) scale.

The statistics measured from the auditory model include marginal moments and pair-wise correlations (Portilla and Simoncelli, 2000; McDermott and Simoncelli, 2011). The included texture statistics are similar to those described in McDermott and Simoncelli (2011). They were computed from the envelope of the cochlea channels, including the first- and second-order modulation filters, and were measured over texture excerpts of several seconds. Examples of the statistics for three textures (insect swarm, campfire, and small stream) measured from the auditory texture model (Figure 1A) are shown in Figure 2.

The envelope statistics include the mean (μ), coefficient of variance ($\frac{\sigma^{2}}{\mu^{2}}$), skewness (η), and kurtosis (κ), and represent the first four marginal moments, defined as:

$$\begin{array}{l} {\mu_{n}=\bar{\vec{x}}}_{\text{n,}}\\ \frac{\sigma_{n}^{2}}{\mu_{n}^{2}}=\frac{\bar{{\left({\vec{x}}_{n}-\mu_{n}\right)}^{2}}}{\mu_{n}^{2}},\\ \eta_{n}=\frac{\bar{{\left({\vec{x}}_{n}-\mu_{n}\right)}^{3}}}{\sigma_{n}^{3}},\\ \kappa_{n}\;=\frac{\bar{{\left({\vec{x}}_{n}-\;\mu_{n}\right)}^{4}}}{\sigma_{n}^{4}}, \end{array}$$

where *n* is the cochlear channel of *x*. Pair-wise correlations were computed as a cross-covariance with the form:

$$c_{mn}=\frac{\bar{\left({\vec{x}}_{m}-\;\mu_{m}\right)\left({\vec{x}}_{n}-\;\mu_{n}\right)}}{\sigma_{m}\sigma_{n}},$$

where *m* and *n* are the cochlear channel pairs. The final statistic captures envelope phase:

$$c_{jk}=\frac{\bar{{\vec{d}}_{k}^{*}{\vec{a}}_{j}}}{\sigma_{k}\sigma_{j}},\;d_{k}=\frac{a_{k}^{2}}{\left\|a_{k}\right\|},\;{\vec{a}}_{k}={\vec{b}}_{k}+\;iH\left({\vec{b}}_{k}\right),$$

where *j* and *k* are the modulation channel pairs of *b*, ***H*** is the Hilbert transform, and * is the complex conjugate.

#### First level statistics

The first level of statistics were measured on the cochlear envelopes of the auditory texture model (Figure 1). The marginal moments (M) describe the distribution of the individual subbands (Figure 2A) and capture the overall level as well as the sparsity of the signal (Field, 1987). The correlation statistics (C) capture how neighboring signals co-vary. The correlation statistics are measured between the eight neighboring cochlear channels (Figure 2B). There are 372 statistics measured at the cochlear stage of the auditory model (*M* = 128, and *C* = 236).

#### Second level statistics

The second level statistics were measured on the first-order modulation bands (Figure 1) and include the coefficient of variance (M^1^P, Figure 2C), the correlation measured across cochlear channels and first-order modulation channels (MC1, Figure 2D), and the correlation measured across modulation channels for the first-order modulations (MC2, Figure 2E). Because the outputs of the modulation filters have zero mean, the variance effectively reflects a measure of the modulation channel power. The variance was measured for cochlear channels that have a center frequency at least four times that of the modulation frequency (Dau et al., 1997). The modulation correlations measured across cochlear channels (MC1) reflect a cross-covariance measure. The correlation was measured for two neighboring cochlear channels. The modulation correlation measured across modulation rates (MC2) included phase information and was computed for octave-spaced modulation frequencies. The number of statistics considered in the modulation domain was 1,258 (M^1^P = 646, MC1 = 408, and MC2 = 204).

#### Third level statistics

The last analysis stage was conducted on the second-order modulation envelope bands (Figure 1), where the modulation power was measured for each band (M^2^P, Figure 2F). This analysis stage extends beyond the model of McDermott and Simoncelli (2011) to capture second-order modulations (Lorenzi et al., 2001b). The power was measured for first-order modulation rates that are at least twice that of the second-order modulation rate. The 2nd-order modulation power required the largest overall number of statistics (M^2^P = 3,400).

### Synthesis system

**Figure 3:**
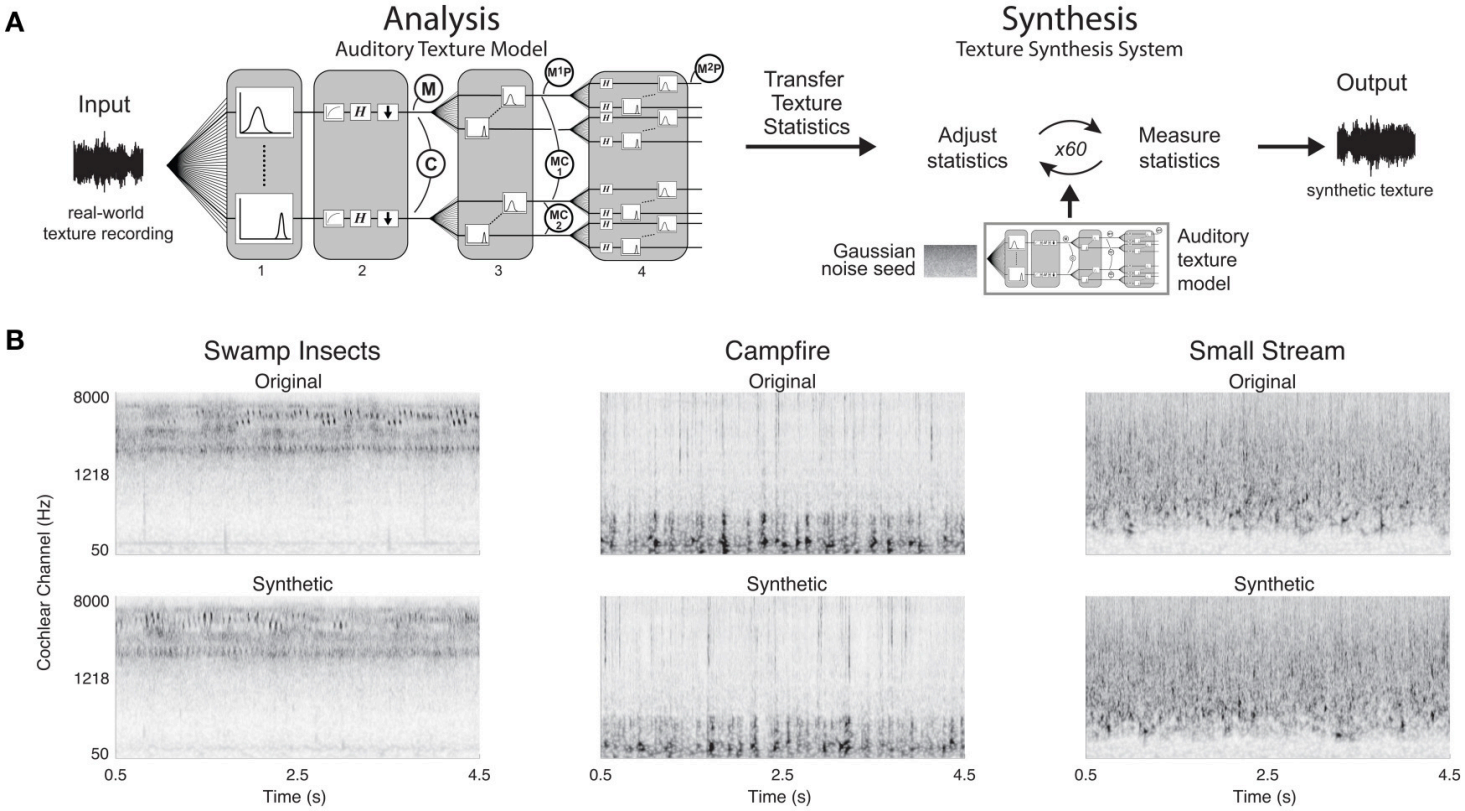
Texture synthesis system and synthetic examples. **(A)** Texture synthesis is accomplished by measuring statistics from a real-world texture recording at different stages of the auditory texture model. The statistics are then passed to the synthesis system that adjusts the statistics of a Gaussian noise seed to match the input statistics. The iterative process outputs a synthetic texture with the same time-averaged statistics as the real-world texture recording. **(B)** Original real-world texture recordings and their synthetic counterparts. The synthetic textures were generated with a complete set of texture statistics. Example audio files corresponding to the original and synthetic spectrograms can be found in the Supplementary Material (Swamp Insects: Audio files 1, 2; Campfire: Audio files 3, 4; Small Stream: Audio files 5, 6).

The synthesis of sound textures was accomplished by modifying a Gaussian noise seed to have statistics that match those measured from a real-world texture recording (Portilla and Simoncelli, 2000; McDermott and Simoncelli, 2011). The original texture recording was decomposed using our biologically motivated auditory model where the texture statistics were measured. The statistics were then passed to the synthesis algorithm which imposed the measured statistics on the decomposed Gaussian noise signal. The modified signals were reconstructed back to a single-channel waveform. A schematic of the synthesis system can be seen in Figure 3A.

The imposition of texture statistics on the noise input was achieved using the LM-BFGS variant of gradient descent (limited-memory Broyden–Fletcher–Goldfarb–Shanno). The noise signal was decomposed to the second-order modulation bands, where the power statistics were imposed. The bands were then reconstructed to the first-order modulation bands, and the modulation power and correlation statistics were imposed. The modulation bands were then reconstructed to the cochlear envelopes, where the marginal moments and pair-wise correlations statistics were imposed. Lastly, the cochlear envelopes were combined with the fine-structure of the noise seed and the cochlear channels were resynthesized to the single channel waveform.

The synthesis process requires many iterations in order to attain convergence for each of the texture statistics due to the reconstruction of the subbands and tiered imposition of statistics. The reconstruction of the filterbanks modified the statistics of each subband due to the overlap in frequency of neighboring filters. The reconstruction from the cochlear envelopes to the cochlear channels was also affected by the combination of the envelope and fine structure. In addition, the texture statistics were modified at 3 layers (cochlear envelopes, 1st-order modulations, and 2nd-order modulations) of the auditory model, and the modification at each level had an impact on the other two. Due to these two factors, an iterative process for imposing texture statistics was required.

The synthesis was deemed successful if the synthetic texture statistics approached those measured from the original real-world texture recoding. The convergence was evaluated based on the signal-to-noise ratio (SNR) between the synthetic and original texture statistics (Portilla and Simoncelli, 2000). When the synthesis process reached an SNR of 30 dB or higher across the texture statistics, the process ended, generating a synthetic texture. The system also had a maximum synthesis iteration limit of 60. However, the convergence criterion was often met within 60 iterations. The cochleograms of the original and synthetic textures are shown in Figure 3B.

### Texture synthesis system validation

**Figure 4:**
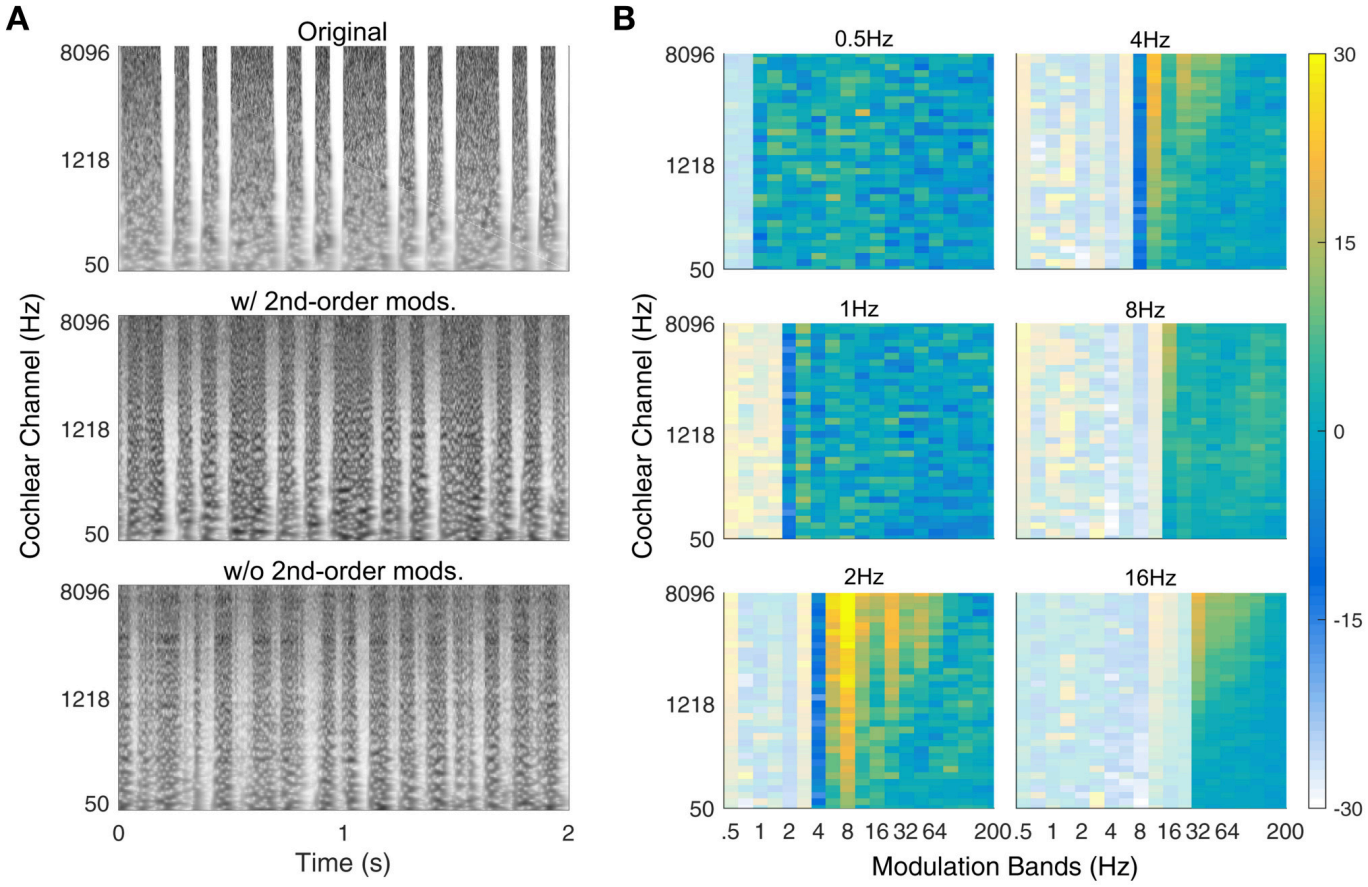
Verification of second-order texture synthesis. **(A)** Spectrogram of example rhythmic (second-order modulated) noise bursts with 500 ms repetition pattern. The upper panel shows the original sound, the middle panel shows the synthetic version with second-order modulation texture statistics (w/ 2nd-order mods.) and the bottom panel shows the synthetic version without second-order modulation texture statistics (w/o 2nd-order mods.). **(B)** Second-order modulation power statistics. The 500 ms period is reflected in the majority of power held within the 2 Hz 2nd-order modulation band (lower-left panel). Example audio files corresponding to the spectrograms can be found in the Supplementary Material (Original: Audio file 7; w/ 2nd-order mods.: Audio file 8; w/o 2nd-order mods.: Audio file 9).

The proposed auditory texture model and adjoining synthesis system were validated with a second-order amplitude modulated signal identified by McDermott and Simoncelli (2011). The signal was generated by applying a binary mask to a Gaussian noise carrier. The mask contained a long noise burst (*t* = 0.1875s or $\frac{3}{16}$ s), followed by two short noise bursts (*t* = 0.0625s or $\frac{1}{16}$ s) that were repeated every 500 ms (see Figure 4A, upper panel). The stimulus has a second-order modulation of 2 Hz, generated by the interaction between two first-order modulations at 6 and 8 Hz.

### Psychophysical experiments

The listeners were recruited from a university specific job posting site. The listeners completed the required consent form and were compensated with an hourly wage for their time. All experiments were approved by the Science Ethics Committee for the Capital Region of Denmark.

The listeners performed the experiment in a single-walled IAC sound isolating booth. The sounds were presented at 70 dB SPL via Sennheiser HD 650 headphones. The playback system included an RME Fireface UCX soundcard and the experiments were all created using Mathworks MATLAB and the PsychToolBox (psychtoolbox.org) software.

The synthetic textures used in experiments 1 and 2 were generated in 5-s long samples. Multiple exemplars were generated for each texture. Each exemplar was created using a different Gaussian noise seed such that no sample was identical in terms of the waveform, but had the same time-averaged texture statistics. Four-second long excerpts were taken from the middle portion of the texture samples with a tapered cosine (Tukey) window with 20-ms ramps at the onset and offset.

#### Experiment 1—texture identification

Each trial consisted of a 4-s texture synthesized from subsets of texture statistics that were cumulatively included from the cochlear envelope mean to the 2nd-order modulation power. The listeners were required to identify the sound from a list of 5 label descriptors. The experiment consisted of 59 sound textures. The textures were divided into 5 texture groups, defined by the authors: animals, environment, mechanical, human, and water sounds. The list of 4 incorrect labels for each texture was selected from different texture groups. There were 7 conditions per texture (6 synthetic and 1 original) and 413 trials per experiment. Eleven self-reported normal-hearing listeners participated in the experiment (6 female, 23.3 mean age).

#### Experiment 2—modulation processing model comparison

Each trial consisted of three intervals; the original real-world texture recording, a synthetic texture generated from the above-mentioned texture synthesis system (reference), and a synthetic texture generated from a modified version of the auditory model. The real-world texture was presented first. Textures generated from the reference system and a modified auditory model were then presented in intervals 2 and 3, where by the order of presentation was randomized. Each interval was 4 s long with an inter-stimulus-interval of 400 ms. The listeners were asked to select the interval that was most similar to the real-world texture recording. The same 59 textures were used in the experiment, presented in 236 trails. Eleven self-reported-normal hearing listeners participated in the experiment (7 female, 24.2 mean age).

Synthetic textures generated from a reference auditory model and four alternate auditory models were included in the experiment. The reference model is described in Figure 1, including texture statistics measured from the cochlear envelope, 1st- and 2nd-order modulation bands. The first alternate model removed the 2nd-order modulation bands, and was in principle similar to that of McDermott and Simoncelli (2011). The second alternate model removed the 2nd-order modulation bands and replaced the half-octave spaced 1st-order modulation filterbank by an octave-spaced variant. Octave-spaced modulation selectivity has been suggested in several models of auditory perception (Dau et al., 1997; Jorgensen and Dau, 2011). The third alternate model removed the 2nd-order modulation bands and substituted the half-octave spaced modulation filterbank with a low-pass filter of 150 Hz. The low-pass characteristic of amplitude modulation perception has been proposed, and here we used a model that preserves the sensitivity to modulation rates but lacks the selectivity of the filterbank model (Kohlrausch et al., 2000; Joris et al., 2004). The fourth alternate model also removed the 2nd-order modulation bands and substituted the half-octave spaced modulation filterbank with a low-pass filter with a cutoff frequency of 5 Hz. The sluggishness of the auditory system to amplitude modulation perception is reflected in the heightened sensitivity to slow modulation rates (Viemeister, 1979; Dau et al., 1996).

#### Experiment 3—second-order modulation discrimination

Each trial consisted of three 2-s intervals. The listeners performed an odd-one-out experiment, where they were instructed to identify the interval (first or last) that was different from the other two. The stimulus sets described below were evaluated in separate experiment blocks. Twelve self-reported-normal hearing listeners participated in the experiment (3 female, 23.0 mean age).

The first stimulus set was generated from second-order amplitude modulated white noise using the following equation:

$$s\left(\;t\right)=\left(1+\left(0.5+\sin\left(2\pi f_{m1}t+\;\phi \right)\right)*\sin\left(2\pi f_{m2}t\right)\right)*n\left(\;t\right),$$

where *f*_*m*1_ is the first modulator, *t* is time, ϕ is the phase of the first modulator, ***f***_***m*2**_ is the second modulator, and *n*(*t*) is the Gaussian noise carrier. *f*_*m*1_ had a modulation frequency of 2, 4, 8, 16, 32, or 64. *f*_*m*2_ had a modulation rate of *f*_*m*1_[0.1, 0.13, 0.17, 0.22, 0.28, 0.36, 0.46, 0.60, 0.77, *or* 1.00]. ϕ was randomized for each trial. The exemplars were 5 s in duration. Two intervals were sampled from the first 2 s, and the “odd” interval was sampled from the last 2 s. Each condition was repeated 4 times, for a total of 240 trials.

The next stimulus set used second-order amplitude modulated white noise generated from a combination of *f*_*m*1_ and *f*_*m*2_ pairs, creating a complex amplitude modulated signal. Each stimulus was created using the six *f*_*m*1_ frequencies, each paired with a corresponding *f*_*m*2_ frequency that was randomly selected from the list of 10, modulating the same white noise seed. The six second-order modulated signals were then summed to create one stimulus. The exemplars were 5 s in duration. Two intervals were sampled from the first 2 s, and the “odd” interval was sampled from the last 2 s. There were 48 stimuli presented, one per trial.

The final stimulus set was composed of sound textures generated with the complete set of texture statistics, including second-order amplitude modulation power. The 59 textures used in experiments 1 and 2 were used in this experiment. The exemplars were 5 s in duration. Two intervals were sampled from the first 2 s, and the “odd” interval was sampled from the last 2 s. There were 59 trials in total.

## Results

The auditory texture model proposed in the present study includes frequency-selective filtering (in the audio-frequency domain) as well as a cascade of amplitude modulation filterbanks to capture time-averaged amplitude modulations and simple rhythmic structure. The model was combined with a sound synthesis system to generate synthetic textures that were then examined in several behavioral listening experiments. The results show three main findings: (1) the model captures simple rhythmic structure by way of second-order amplitude modulation analysis, (2) the inclusion of second-order amplitude modulation analysis contributes to the recognition of the synthetic textures, and (3) second-order amplitude modulations in textures may be perceived using time-averaged summary statistics.

### Synthesis verification for second-order modulations

Although, the second-order texture statistics varied across textures, it was unclear how the synthesis process would perform in creating new sound examples. To test this, we used a second-order amplitude modulation signal identified by McDermott and Simoncelli (2011) that has a salient rhythmic structure. Figure 4A shows the original sound (top), a synthetic version with second-order modulation analysis (middle) and a synthetic version without second-order analysis (bottom). The synthetic sound generated from texture statistics that included second-order amplitude modulation analysis captured the rhythmic pattern of the original sound, whereas the version without second-order analysis failed to capture the rhythmic structure even though the duration of the noise bursts is comparable to that in the original sound. The successful synthesis of the rhythmic sound suggests that the cascaded modulation filterbank analysis can capture rhythmic structure.

The second-order amplitude modulation statistics for the example rhythmic sound are shown in Figure 4B. The majority of the modulation power can be found in the 2 Hz second-order modulation channel (bottom left panel) across several first-order modulation rates. For a relatively simple rhythmic sound, there is considerable modulation power across frequencies. This is primarily due to amplitude modulation interactions between the modulation frequencies and the broadband (Gaussian) noise carrier. If a second-order amplitude modulated tone was used instead of the noise with its intrinsic modulations, the modulation power would be relegated entirely to the 2-Hz band.

### Texture perception: identification and preference

**Figure 5:**
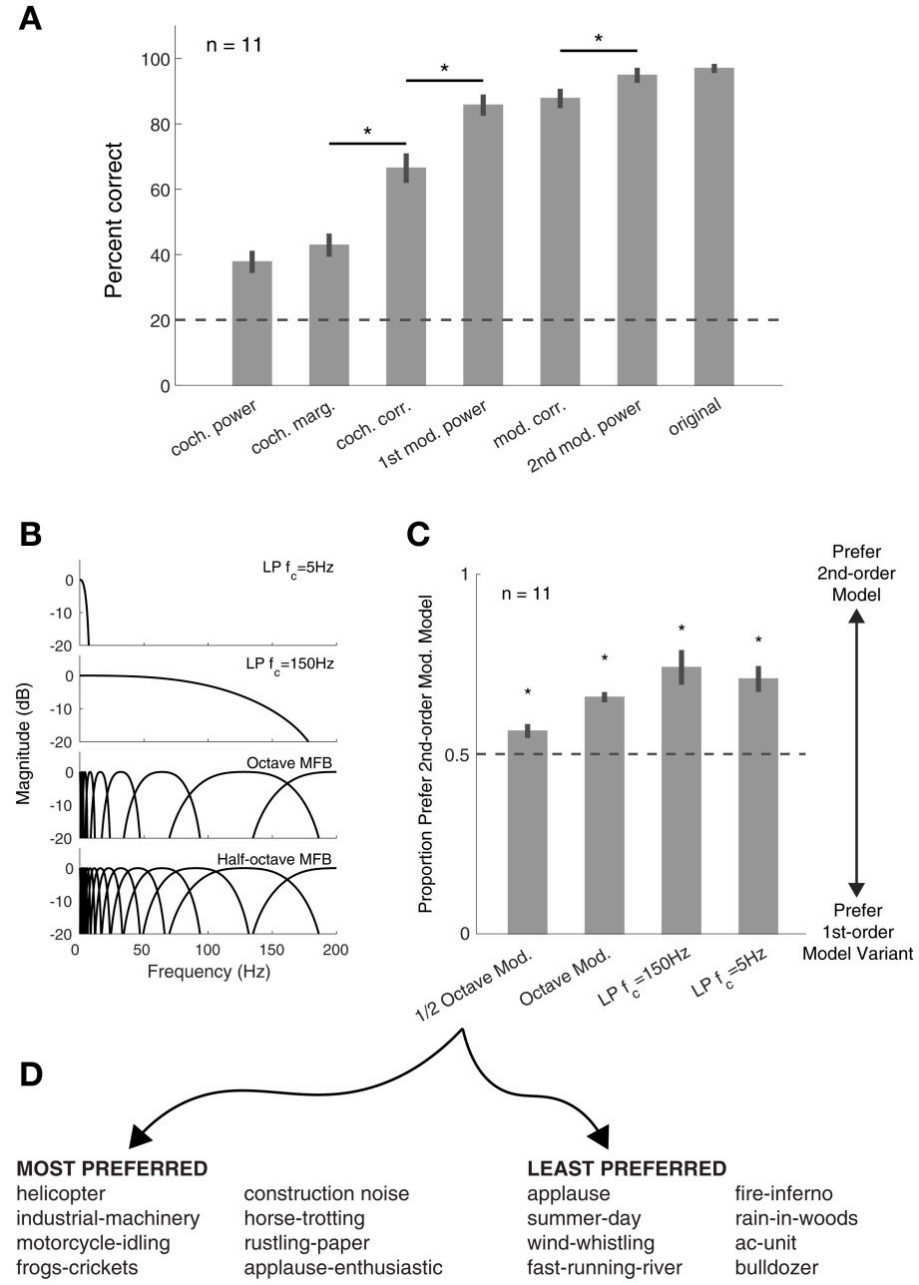
Synthetic texture identification and preference tasks. **(A)** Identification of sound textures improves with the inclusion of more statistics. Asterisks denote significant differences between conditions, *p* < 0.01 (paired *t*-tests, corrected for multiple comparisons). Error bars here and elsewhere show the standard error. Dashed lines here and elsewhere show chance performance. **(B)** Modulation filter(bank) structure used in the listening experiments. For low-pass (LP) conditions, only the statistics of the signal in the passband were modified. **(C)** Sounds synthesized with the 2nd-order modulation statistics were preferred over all other auditory texture models. Asterisk denotes significance from chance (*p* < 0.01). **(D)** Eight most preferred (left) and least preferred (right) textures from experiment 2, relative to first-order modulation filterbank model (half-octave spacing).

Our first behavioral experiment investigated the ability of listeners to identify sound textures generated from subsets of statistics. Listeners were presented with a 4 s texture and asked to identify the sound from a list of 5 text label descriptors. The textures synthesized with the cochlear envelope power resulted in low performance, but the performance increased with the inclusion of higher-order texture statistics and approached that of the original real-world texture recording when second-order amplitude modulation statistics were used [Figure 5A; *F*_(6, 49)_ = 123.51, *p* < 0.0001]. The results suggest that listeners benefited from the addition of second-order amplitude modulation analysis to the auditory texture model.

Next, we were interested in how synthetic textures generated with alternate amplitude modulation processing models compared to our auditory texture models. To investigate this, we generated textures from four models that included only the first-order amplitude modulation analysis (Figure 5B). The results show that our auditory texture model, with second-order amplitude modulation analysis, was preferred over all other model variants (Figure 5C; *p* < 0.01 relative to chance). Notably, the inclusion of second-order modulation analysis yielded a modest yet significant improvement over the half-octave spaced first-order modulation, which is comparable to that developed by McDermott and Simoncelli (2011). The results from the preference experiment revealed which textures benefited most from second-order amplitude modulation analysis. Figure 5D shows a list of the top 8 most preferred and least preferred textures measured between the half-octave spaced filterbank and our auditory texture model. The list includes a broad range of sounds, from mechanical/machine noises to animal/insect sounds. The least preferred textures reveal sounds which may not depend greatly on amplitude modulation texture statistics (i.e., cochlear envelope marginal moments and pair-wise correlations).

**Figure 6:**
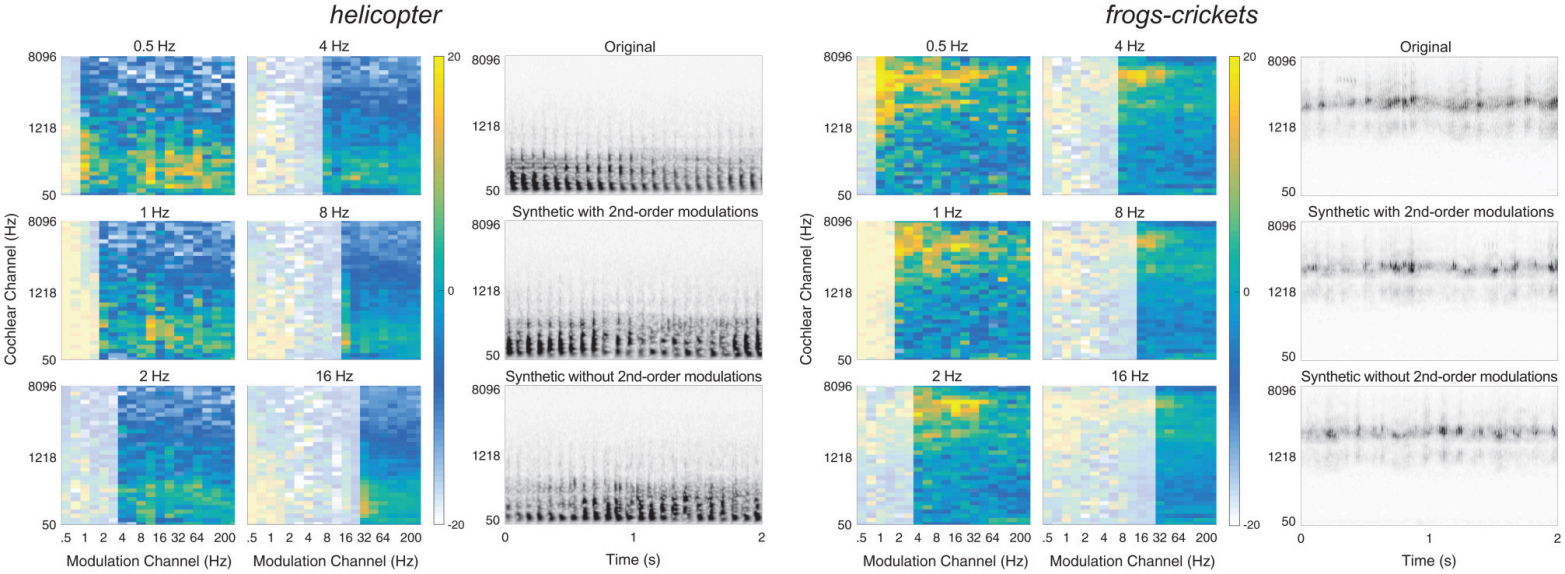
Textures that benefit from second-order modulation statistics. Two example textures from the preferred list: Helicopter (left) and frogs-crickets (right). The left panel shows the second-order modulation statistics for six selected bands. The right panel shows the spectrogram of the original texture (top) and the synthetic texture with second-order modulation statistics (middle) and without second-order modulation statistics (bottom). Example audio files corresponding to the spectrograms of the original, synthetic with 2nd-order modulations, and without 2nd-order modulations can be found in the Supplementary Material (helicopter: Audio files 10–12; frogs-crickets: Audio files 13–15).

Two example textures, *helicopter* and *frogs-crickets*, are shown in Figure 6. For each texture, the left panel shows the 2nd-order modulation texture statistics for selected bands and the right panel shows the original and synthetic texture cochleograms. Notably, the second-order amplitude modulation power differs between the two textures, suggesting that the additional analysis contributes to sound texture recognition.

### Second-order modulation discrimination

To examine if second-order amplitude modulations are processed by the auditory system similarly to textures, i.e., integrated over modest time windows of a few seconds, or if the auditory system has the temporal acuity to identify and discriminate second-order modulations with higher precision, a set of discrimination experiments was performed where synthetic sound textures were compared to artificial control stimuli generated from amplitude modulated Gaussian noise. Listeners performed a three-interval odd-one-out experiment, where they were asked to identify whether the first or last interval was different from the other two. The experiments covered three stimulus groups: rate-specific second-order amplitude modulations, complex second-order amplitude modulation noise from a set of modulation rates, and synthetic sound textures generated using second-order amplitude modulation statistics.

**Figure 7:**
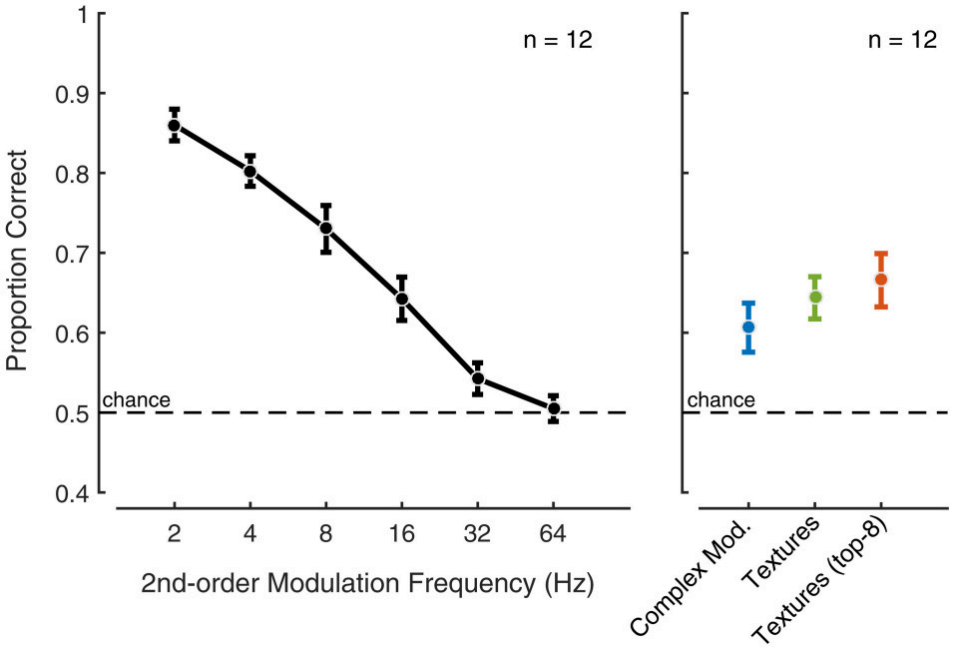
Second-order amplitude modulation and texture exemplar discrimination. The black symbols show the response to second-order amplitude modulated Gaussian noise exemplar discrimination as a function of modulation rate. Error bars indicate the standard error. The blue symbol indicates exemplar discrimination performance for complex second-order amplitude modulated Gaussian noise. The green symbol indicates exemplar discrimination performance for synthetic sound textures that include all indicated texture statistics (including second-order amplitude modulation statistics). The red symbol indicates exemplar discrimination performance for top-8 synthetic (Experiment 2) sound textures that include all indicated texture statistics.

The first experiment included second-order amplitude modulations of increasing rate from 2 to 64 Hz. The results showed that, at low rates, the listeners have the ability to discriminate modulated noise exemplars (Figure 7—left panel). The performance decreased with increasing modulation rate and approached chance level for modulation rates above 16 Hz. For these control stimuli, the results suggest that the auditory system may have access the modulation phase for rates 16 Hz and below.

The discriminability of the complex modulated Gaussian noise and the synthetic texture was poor (Figure 7—right panel) compared to the low modulation rates considered in the previous experiment. This suggests that, for texture sounds, access to the modulation phase is limited in the auditory system. Isolating the top eight most preferred textures from Experiment 2 revealed comparable performance to the complete set of textures. The performance observed for sound textures in a similar odd-one-out discrimination task was comparable to that reported in McDermott et al. (2013) for an interval duration of about 2 s. Collectively, the results suggest that textures, including those that benefit from second-order modulation analysis, may be perceived using time-average statistics, whereas the auditory system appears to retain more temporal detail for our second-order modulation control stimuli for rates below 16 Hz.

## Discussion

The perception of sound texture can be characterized by a set of time-averaged statistics measured from early auditory representations. We extended the auditory texture model of McDermott and Simoncelli (2011) to account for simple rhythmic structures in sound textures via a cascade of amplitude modulation filterbanks. The auditory texture model was coupled with a sound synthesis system to generate texture exemplars from the statistics measured at different stages of the model. The synthetic stimuli were first used in a texture identification experiment, where the listeners' ability to recognize a texture improved with the inclusion of the subgroups of statistics. We found that the performance obtained using the second-order amplitude modulation analysis approached that of the original real-world texture recordings and was higher than the performance obtained using only a first-order amplitude modulation analysis (Experiment 1). We also generated synthetic textures from alternate auditory models of amplitude modulation sensitivity. The synthetic textures were used in a preference task, where listeners' preferred sounds synthesized using second-order amplitude modulation over all other model variants (Experiment 2). Lastly, we performed an experiment focusing on second-order amplitude modulation perception in a discrimination task. The listeners' ability to discriminate second-order modulation sound exemplars decreased with increasing modulation rate, and complex second-order modulated Gaussian noise and synthetic textures appear to be perceived using a time-averaging mechanism (Experiment 3).

### Amplitude modulations in texture perception

The auditory texture model described by McDermott and Simoncelli (2011) included a biologically plausible first-order modulation filterbank operating on individual cochlear channel envelopes. The textures synthesized with this model produced many compelling textures, including sounds generated from machinery (e.g., helicopter, printing press) with relatively uniform short-time repetitions as well as environmental sounds (e.g., wind, ocean waves) with variable slow modulations. Our texture model built upon this work and provided further evidence for the importance of modulation selectivity in sound texture perception. For first-order modulation analysis, the results from the preference task (Experiment 2) demonstrated that using half-octave spaced modulation filterbank yields the best performance out of the model variants. The model has a slightly higher selectivity than has that reported in earlier models (Dau et al., 1997). One reason may be that the selectivity of the auditory system for natural sounds, such as textures, may be slightly different than that for artificial stimuli used to identify the auditory systems' modulation tuning curves and selectivity. Another possible explanation is that natural sounds do not conform to octave spaced modulation frequencies, and if the modulation power in a natural sound has a maximum between two modulation bands with fixed center frequencies, the synthetic sounds vary to a greater degree from the original real-world recording.

The results from the preference experiment also identified which textures were most improved (preferred) by the inclusion of the second-order modulation analysis. These textures tended to have higher first-order modulation power, but did not appear to possess obvious common feature. Some sounds, such as the helicopter, had low second-order modulation power while others, such as the frogs-crickets, had high second-order modulation power. Also, the second-order modulation power error between the first-order model and the second-order model did not tend to be higher for these textures. Intuitively, there may be aspects of first-order modulations that are captured by our model, such as mediating the modulation depth in our time-averaged measurements. However, this was difficult to reveal with our natural texture stimuli.

### Model architecture and statistics

There might be several auditory model architectures that can successfully capture rhythmic structure in sound textures. Our proposed model, using a cascade of modulation filterbanks, seems to provide a compelling approach, as it is relatively intuitive and straight forward to implement in the already established texture analysis-synthesis framework. Another option, however, would be the “venelope” model proposed by Ewert et al. (2002) which used a side-chain analysis to measure the second-order amplitude modulations. In this model, the second-order modulations are extracted from the cochlear envelope and analyzed using a single modulation filterbank. The “venelope” model is more efficient than our cascaded model and there is some evidence to suggest that second-order modulations are processed in the auditory system using the same mechanism as the first-order modulation (Verhey et al., 2003). However, the cascaded modulation filterbank model considered in this study can capture simple rhythmic structure and provided an easier means to reconstruct the filters and thus synthesize textures.

Our approach to modeling of the auditory system, based on audio-frequency and amplitude- modulation-frequency selective filtering, is consistent with biological evidence from the mammalian auditory system (Ruggero, 1992; Joris et al., 2004; Rodríguez et al., 2010). This is found in the auditory-inspired filter structure for both cochlear channels and modulation-selective channels, which culminated in a cascade of filterbanks with intermediate envelope extraction using the Hilbert transform. A similar hierarchical processing architecture has also been well-defined by Mallat and colleagues as scattering moments (Mallat, 2012; Bruna and Mallat, 2013). The scattering moments have been shown to capture a wide range of structure in natural stimuli (Andén and Mallat, 2011, 2012, 2014), in addition to being used for sound texture synthesis (Bruna and Mallat, 2013).

A consequence of the cascaded filterbank model proposed here is that the number of statistics required to capture the auditory feature increases with each layer. This is predominantly the case for the second-order modulation analysis, where we measure 3,400 parameters, which increases the number of texture statistics by a factor of ~3 as compared to the model of McDermott and Simoncelli (2011). It may be possible to optimize the number of parameters by identifying which modulation rates are most significant for texture perception. Alternate models, such as the “venelope” model of Ewert et al. (2002), could reduce the number of parameters needed to capture the second-order amplitude modulation. Although the additional model layer increased the number of statistics, the representation is moderately compact as the statistics are computed as time-averages of the signal.

An alternate approach to representing textures via statistics, is to learn efficient representations from the stimuli themselves. This approach has been shown to be useful for identifying sparse representations of natural stimuli from hierarchical models (Karklin and Lewicki, 2005; Cadieu and Olshausen, 2009). The higher-order structure of natural sounds, such as environmental textures, has also been explored to uncover their possible neural representation (Młynarski and McDermott, 2017). These methods come with their own complications and limitations, however may be a useful avenue for identifying more efficient representations than the texture model of McDermott and Simoncelli (2011) or the one outlined in the present study.

### Temporal regularity in texture perception

Sounds textures have been defined as the superposition of many similar acoustic events, therefore it was not obvious *a priori* that sounds with temporal regularities would be perceived in the same way—as time-averages of sensory measurements. Temporal patterns are important for sound perception, and their contribution has been investigated in terms of auditory streaming (Bendixen et al., 2010; Andreou et al., 2011). In addition, sensitivity to temporal regularities in the auditory system has also been shown in complex listening environments (Barascud et al., 2016). Our results show that second-order modulation statistics vary across textures, and the inclusion of this second modulation analysis generated modest improvements in the perceived quality of the synthetic textures. Textures generated with second-order amplitude modulation analysis seemed to result in similar discriminability, suggesting that the features captured by the cascaded modulation filterbank model may be perceived via a similar time-averaging mechanism that has been proposed for more noise-like textures.

### Relationship to visual texture perception

One of the interesting ideas about texture perception is that of a unified representation across sensory modalities. Textures have been investigated in the visual system (Julesz, 1962; Portilla and Simoncelli, 2000; Freeman and Simoncelli, 2011), the somatosensory system (Connor and Johnson, 1992) and the auditory system (Saint-Arnaud and Popat, 1995; McDermott and Simoncelli, 2011). Of particular relevance to our work is how the sound texture synthesis system proposed by McDermott and Simoncelli (2011) is comparable in processing structure and analysis to that presented by Portilla and Simoncelli (2000) for visual textures. In both models, the input signal is processed by layers of linear filtering and envelope extraction, while the texture analysis statistics, which are primarily composed of marginal moments and pair-wise correlations, are also similar between the two models. Our model of cascaded filterbanks also overlaps with other models of the image texture perception (Wang et al., 2012). It therefore seems valuable to look across sensory modalities for shared perceptual spaces (Zaidi et al., 2013).

Our investigation of second-order modulation analysis in sound texture perception may also be relatable to spatial texture patterns, or maximally regular textures, in the visual system. Kohler et al. (2016) showed a neural sensitivity to image texture patterns that repeat in space. Our work is also indicative of sound texture pattern sensitivity in time. Previous work in both sound and image texture perception has also made the comparison of perceptual pooling over time and space, respectively (Balas et al., 2009; Freeman and Simoncelli, 2011; McDermott et al., 2013). Conceptually, the apparent texture time-averaging in audition draws compelling parallels to the spatial averaging observed in visual texture perception.

### Implications and perspectives

In this study, we investigated the significance of second-order amplitude modulations in natural sound texture perception. The generation of synthetic sound textures using a cascade of modulation filterbanks appears to contribute positively to the perception of texture. We also observed that the auditory system is sensitive to specific rates of second-order modulations, showing heightened acuity to isolated modulations for rates below 16 Hz. Future experiments would be useful to understand the role of temporal regularity in texture at different modulations rates and spectral frequencies. In addition, such stimuli could be useful to understand the perception of texture in complex auditory scenes, such as the perceptual segregation of speech in the presence of different types of background textures.

## Ethics statements

This study was carried out in accordance with the recommendations of Danish Science-Ethics Committee (Den Nationale Videnskabsetiske Komité), Capital Region Committees (De Videnskabsetiske Komitéer for Region Hovedstaden) with written informed consent from all subjects. All subjects gave written informed consent in accordance with the Declaration of Helsinki.

## Author contributions

RM performed the experiments and analysis. All authors designed the experiments, interpreted the results, and wrote the paper.

### Conflict of interest statement

The authors declare that the research was conducted in the absence of any commercial or financial relationships that could be construed as a potential conflict of interest.

## Supplementary material

Audio file 1 **Figure 3B (Swamp Insects–Original)**.

Audio file 2 **Figure 3B (Swamp Insects–Synthetic)**.

Audio file 3 **Figure 3B (Campfire–Original)**.

Audio file 4 **Figure 3B (Campfire–Synthetic)**.

Audio file 5 **Figure 3B (Small Stream–Original)**.

Audio file 6 **Figure 3B (Small Stream–Synthetic)**.

Audio file 7 **Figure 4A (Original)**.

Audio file 8 **Figure 4A (w/ 2nd-order mods.)**.

Audio file 9 **Figure 4A (w/o 2nd-order mods.)**.

Audio file 10 **Figure 6 (helicopter–Original)**.

Audio file 11 **Figure 6 (helicopter–Synthetic with 2nd-order modulations)**.

Audio file 12 **Figure 6 (helicopter–Synthetic without 2nd-order modulations)**.

Audio file 13 **Figure 6 (frogs-crickets–Original)**.

Audio file 14 **Figure 6 (frogs-crickets–Synthetic with 2nd-order modulations)**.

Audio file 15 **Figure 6 (frogs-crickets–Synthetic without 2nd-order modulations)**.
